# Factors Affecting the Choice and Level of Adaptation Strategies Among Smallholder Farmers in KwaZulu Natal Province

**DOI:** 10.3390/su17020488

**Published:** 2025-01-10

**Authors:** Merishca Naicker, Denver Naidoo, Simphiwe Innocentia Hlatshwayo, Mjabuliseni Simon Ngidi

**Affiliations:** 1 African Centre for Food Security (ACFS), School of Agriculture Earth and Environmental Sciences, University of KwaZulu-Natal, Pietermaritzburg 3201, South Africa; 2 Department of Agricultural Extension and Rural Resources Management, School of Agriculture Earth and Environmental Sciences, University of KwaZulu-Natal, Pietermaritzburg 3201, South Africa; 3 Centre for Transformative and Agricultural Food Systems (CTAFS), School of Agriculture Earth and Environmental Sciences, University of KwaZulu-Natal, Pietermaritzburg 3201, South Africa

**Keywords:** adaptation strategies, smallholder vegetable farmers, KwaZulu Natal, level of adaptation, climate variability

## Abstract

Smallholder vegetable farmers grow diverse crops for family use and surplus sales. These farming activities contribute to enhancing local food security and the economy, but the farmers face challenges like limited resources and climate vulnerability. These smallholder farmers are more susceptible to climate variability and therefore need effective adaptation strategies to mitigate the challenges. This study sought to determine the factors that influence the choice and level of adaptation strategies among smallholder vegetable farmers in KwaZulu Natal Province. Primary data utilized in this study were obtained from 200 participants that were selected through random sampling. The descriptive results indicated that the majority of the farmers experienced climate variability and employed carbon and water-smart agricultural practices. The study employed the Multivariate Probit Model and Count Data Model/GLM Correlation Test to analyze the adaptation strategies and the level of their implementation by the selected vegetable farmers. The first hurdle of the probit model results showed that education level and land size positively and significantly influence smallholder farmers’ adaptation strategies, while marital status, household size, income source, soil type, membership of the association, and supply chain involvement had a significant and negative effect on adoption of adaptation strategies. The results from the second hurdle showed that household size, the total size of land used for vegetable production, sandy, silt, and loam soil had a significant and negative effect on the level of adaptation strategy used, whilst the total size of land owned had a positive and significant impact on the level of adaptation strategy used by the smallholder vegetable farmers. The study concludes that education level and total land size are associated with improved farmers’ climate variability adaptative capacity. The vegetable farmers’ ability to adapt to climate variability challenges was negatively influenced by factors like marital status, household size, and soil type negatively impacted these strategies. The study recommends that the government considerably supports the Climate Smart Agriculture initiatives, such as alternatives like hot houses, training, credit access, and sustainable practices to enhance farmers’ resilience and national food security. These may include but are not limited to alternatives like hot houses, as well as addressing barriers through training, credit access, and sustainable practices to enhance farmers’ resilience and national food security.

## 1 Introduction

Agriculture is a crucial sector that contributes to economic development, social well-being, and environmental sustainability [1,2]. It is a source of food, supports growth, and enhances productivity through cultivation expansion. Productivity and market efficiency of smallholder farmers are improved through modernization [3]. It significantly improves livelihoods and reduces food insecurity among rural households [4]. Agriculture contributes to 19.26% of employment in South Africa, with KwaZulu Natal (KZN) being the second largest contributor [5]. In South Africa, especially KZN agriculture, is a key sector, particularly for smallholder vegetable farmers. Smallholder vegetable farmers refer to those farmers who produce various vegetables (potatoes, cabbages, beetroot, spinach, tomatoes, etc.) mainly for family consumption and sell the surplus. These farmers produce vegetables that are mostly organic and contribute to food and nutrition security. Moreover, smallholder vegetable farmers play a huge role in ensuring local food access and contribute to the local community economy and poverty reduction in KZN. Despite the potential of smallholder farming, they are characterized by limited land access, poor storage facilities, poor infrastructure, and market orientation. Additionally, smallholder vegetable farmers are more susceptible to climate variability as they have poor managerial production skills and limited access to quality irrigation systems. This emphasizes the need for effective adaptation strategies to aid smallholder farmers in KZN [6].

Climate variability poses significant challenges to smallholder farmers in KZN, impacting their livelihoods and agricultural productivity. The vegetables that are grown by smallholder farmers have a low shelf life and are easily perishable; therefore, an increase in temperatures and erratic rainfall patterns result in a negative impact on the production, yield, and storage of the vegetables. However, smallholder vegetable farmers use adaptation strategies that enhance their resilience to climate variability, optimize their use of resources, and incorporate sustainable practices which boost their profits and yields [7]. These adaptation strategies include growing drought-resistant crops, crop diversification, adjusting planting dates, soil and water conservation practices, reducing livestock holdings, planting trees, extension services, remittances, family labor, and small-scale irrigation, mitigating climate variability effects [8–11]. Smallholder farmers benefit from using these adaptation strategies as they are cost-effective, do not require in-depth knowledge, and are easy to implement, though various adaptation strategies exist and are used, most farmers do not utilize them effectively [12]. Also, these adaptation strategies are environmentally friendly, easily accessible, and sustainable.

Smallholder farmers’ adaptation strategies to climate variability are influenced by awareness, resource access, household perception of climate change, awareness of climate-related risks, and perceived impact of climatic events socio-demographic characteristics, and the role of farmer groups and agricultural extension services [13–15]. Other factors include farming practices, information distribution, resource management, and community support. Enhancing the adaptive capacity of smallholder farmers through education, targeted interventions, and policy support is essential for building resilience and sustainable agricultural practices [16,17]. It is therefore important to understand these determining factors for enhancing farmers’ resilience to climate variability and ensuring sustainable agricultural practices [18].

Previous studies by Hirpha et al. [19], Addis and Abirdew [20], and Ikua [21] have examined the factors influencing smallholder farmers’ adaptation strategies in general; yet, none have specifically focused on smallholder vegetable farmers. Yet, the government of South Africa and other non-governmental organizations are strongly advocating for the consumption of vegetables to improve the food and nutrition security profile of the province and the country in general, creating a need to focus on these farmers. In regions such as East Africa, studies like those by Mutekwa et al. [22] in Zimbabwe have explored how vegetable farmers adapt to climate change; yet, there remains a gap in research that investigates the specific adaptation strategies of vegetable farmers in South Africa. In Kenya, the work of Mwangi et al. [23] highlighted how vegetable farmers face unique challenges, including high susceptibility to extreme weather conditions and market volatility, but have not entirely studied the broader socio-economic factors that affect strategy selection.

While previous studies have explored the challenges farmers face in adopting effective adaptation strategies, such as limited knowledge and financial constraints, they have often overlooked the exclusive difficulties faced by smallholder vegetable farmers. These studies highlight external barriers like restricted access to inputs, inadequate infrastructure, and high transaction costs, which prevent farmers from accessing high-value markets. However, there is a notable gap in the literature regarding the specific factors influencing the choice of adaptation strategies and the extent to which these strategies are implemented, particularly for vegetable farmers. The focus on smallholder vegetable farmers is especially significant as vegetables, unlike staple crops, are more vulnerable to climate extremes and require specific adaptation strategies to address both environmental and market-related challenges. This study aims to address this gap by investigating the factors that influence the selection and implementation of adaptation strategies among smallholder vegetable farmers in KwaZulu Natal Province, offering valuable insights into a sector that has received limited attention in existing research.

## 2 Materials and Methods

### 2.1 Description of Study Area

**Figure 1:**
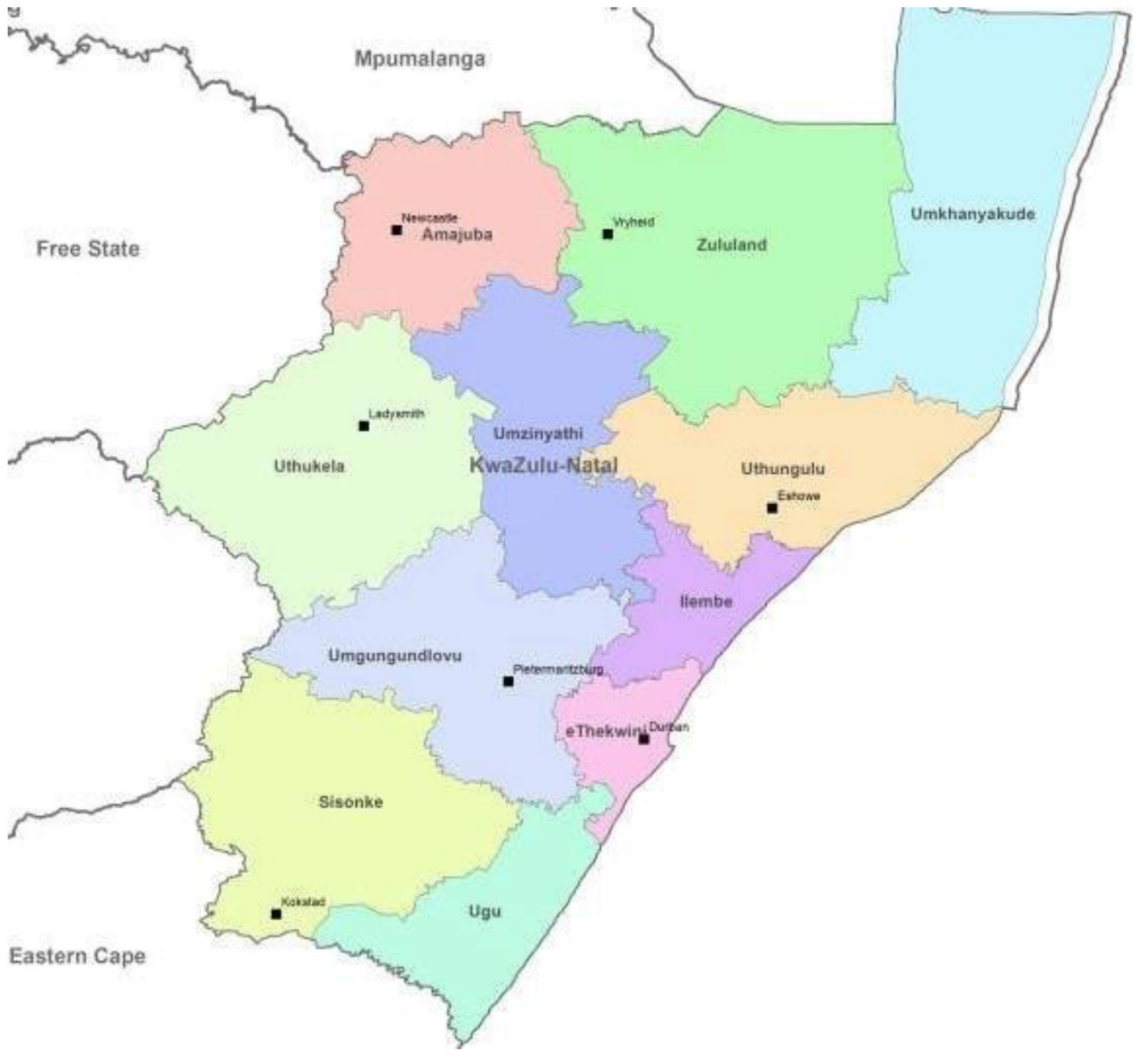
Map of the study area—KwaZulu Natal Province, South Africa (accessed from DAFF: https://www.researchgate.net/publication/346937802/figure/fig1/AS:972334794018817@1608834088348/Map-of-KwaZulu-Natal-district-municipalities-DAFF-nd_Q320.jpg, accessed on 8 July 2024).

This study was conducted in KwaZulu Natal (KZN), a province in South Africa. The province has a diverse topography and experiences high rainfall in January and dry conditions during June [24]. The annual maximum temperature experienced in the province is 24.77 °C and the minimum temperature of 19.7 °C. There is an average of 106.56 mm of precipitation every year. One of the main industries in the province’s rural areas for employment is agriculture. The province has a diverse economy, with industry, tourism, and agriculture being its three main economic areas. The study focused on the entire province and covered all eleven districts as seen in Figure 1 below. The study area was chosen due to the current climatic variability, such as the flash floods and extreme heat, that has altered the agricultural production of smallholder farmers in the region. The effects of the COVID-19 pandemic and the looting have added pressure on the farmers and the quality and quantity of produce [25,26]. The KwaZulu Natal (KZN) province was selected as the study area due to its significant agricultural output, encompassing major crops such as sugarcane, maize, and subtropical fruits, coupled with its documented vulnerability to extreme weather events. To minimize selection bias, the research encompassed smallholder farmers across the entire provincial region.

### 2.2 Data Collection Method

This study applied a quantitative method approach. This approach is particularly useful for hypothesis testing, as it enables researchers to utilize statistical techniques to analyze data and draw evidence-based conclusions [27]. Data collection took place between December 2023 and March 2024. Ethical clearance was obtained from the Human and Social Science Research Ethics Committee (HSSREC)—HSSREC/00005925/2023. The data collected included quantitative methodologies to achieve an understanding of the factors that influence the choice and level of adaptation strategies among smallholder vegetable farmers in KwaZulu Natal Province. These included demographics, socio-economic characteristics, adaptation methods, levels of adaptation methods, and Climate Smart Agricultural practices. The data were not limited to the above-mentioned data.

The questionnaire administered to farmers included both closed and open-ended questions. These were enumerated by the researcher; however, if there were language barriers between the respondent and researcher, Zulu-speaking enumerators administered the questionnaires and focused group discussions. The questionnaires were audio-recorded with the participants’ permission. This practice enhances transparency in research and allows for the replication of studies, contributing to the overall reliability and validity of the research findings [28].

Simple random sampling was employed to ensure a diverse representation of small-holder vegetable farmers across different rural regions of KwaZulu Natal. A 95% confidence interval was employed with a 5% margin for error. This sampling technique is characterized by its simplicity and fairness, as it provides an unbiased way to select participants from a larger population [29]. This method is particularly useful when researchers aim to draw conclusions that apply to the entire population under study [30]. The sample size consisted of 200 smallholder vegetable farmers for the questionnaires, randomly selected from a sampling frame of 2530 farmers. These farmers were chosen because they were registered with local agricultural organizations or market operators in the province. While selecting farmers registered with local agricultural organizations may have excluded the unregistered farmers, these farmers share similar characteristics [31]. This ensured that there was a comprehensive representation of the smallholder farmers in the province.

### 2.3 Data Analysis

Once the fieldwork was completed, the questionnaires were checked for accuracy and completeness. Quantitative models and descriptive statistics were calculated using IBM SPSS (Statistical Package for Social Sciences), version 28; Excel was also used to analyze the quantitative data.

Various statistical and data science techniques were employed in this study. The integration of statistical tools and qualitative modeling techniques has been utilized to effectively manage large amounts of agricultural data, providing insights into complex agricultural processes [32].

The study employed a Multivariate Probit Model to analyze the factors influencing farmers’ choices of multiple adaptation strategies, examining the probability of adopting several strategies simultaneously and considering the interdependence between them. The key variables considered in the ordered probit regression model were gender, age, education level, marital status, household size, number of years farming vegetables, whether farming was the participant’s main source of income, total size of land owned (hectare), total size of land used for farming, type of soil—sand, type of soil—silt, type of soil—clay, type of soil—loam, whether the participant was a member of a farmers’ association/co-operation and involved in any formal or informal food supply chain networks or associations. The application of the multivariate probit model in agricultural research facilitates the identification of factors that influence farmers’ decisions to adopt specific agricultural technologies or practices. These models offer a robust framework for analyzing the complexities of adoption behavior, allowing researchers to uncover the underlying drivers of technology uptake and the interactions between different variables [33,34]. The Multivariate Probit Model was used to model outcomes of multiple binary dependent variables simultaneously, where these outcomes might be correlated. It extends the probit model to multiple equations [33].

Additionally, a Count Data Model/GLM Correlation Test was used to analyze count data, such as the number of adaptation strategies implemented by each farmer. The data followed a normal distribution before the correlation analysis was conducted. Count data models are particularly useful when dealing with discrete variables that represent counts, such as the number of pests in a field or the frequency of a specific event. These models are valuable for analyzing agricultural data that involve non-negative integers and are commonly used in various agricultural studies [35]. Correlation tests were conducted to identify relationships between demographic factors, livelihood variables, and the number of adaptation strategies adopted. Statistical analyses were conducted using software such as Stata, version 18.

Equi dispersion, or the equality of variance and mean, is an assumption made by the Poisson distribution. When the variance is less than the mean, it is called under-dispersion, and when it is greater than the mean, it is called over-dispersion [36]. Using the method used by Abate and Addis [37], let y_i_ denote the number of occurrences at a specific time or exposure period, with a rate given by *µ*_i_. The following equations outline the specifications for the Poisson regression model:

(1) $$\text{P}({\text{Y}}_{\text{i}}={\text{y}}_{\text{i}},\mu )=\frac{{\text{e}}^{-\mu_{\text{i}}\mu^{\text{y}}}}{{\text{y}}_{\text{i}}^{!}},\mu_{\text{i}}>0,i=1,2\ldots n,{\text{ and y}}_{\text{i}}=0,1,2,3\ldots$$

The equation can be represented further by

(2) $$\mu =\exp(\beta_{0}+\beta_{1}{\text{x}}_{1\text{i}}+\beta_{2}{\text{x}}_{2\text{i}}+\ldots +\beta_{\text{k}}{\text{x}}_{ki})$$

In this study, y_i_ represents the value of an event count outcome variable with a mean parameter of *µ*_i_ that occurs during a specific time or exposure period [37]. Assumed to be a non-linear function of the independent variables, *µ*_i_ represents the mean and variance of the Poisson distribution. β_0_ is the intercept of the model, where the coefficients of independent variables are denoted as β_1_; β_2_…β_k_ and the number of explanatory variables [38]. Table 1 indicates the expected outcomes of the variables.

## 3 Results

### 3.1 Descriptive Results

#### 3.1.1 Demographic Characteristics of Smallholder Farmers in KwaZulu Natal

The sociodemographic characteristics of surveyed smallholder farmers are summarized in Table 2 using descriptive statistics. The data show that 85% of the surveyed population relies primarily on farming for their income, while 15% do not. Table 2 illustrates that most participants were male (65%), and the average age group was between 40 and 53 years (30%). Additionally, 50.5% of smallholder farmers had completed secondary education. Age and education levels significantly influence the adaptation strategies of smallholder farmers. Less educated and older farmers are more inclined to use traditional practices and are generally more hesitant to adopt modern strategies to combat climate variability [39].

The most common household size among the respondents was 3–4 people (37.5%), while the least common was 11–12 members (4%). Most of the sample population were household heads (66.5%). According to Table 2, 40.5% of the respondents did not belong to any farmers’ groups, 33% were part of farmers’ associations, and 26.5% were involved in cooperatives. Membership in these groups often exposes farmers to valuable information and increases their likelihood of adopting modern adaptation strategies [40]. Smallholder farmers are crucial to South Africa’s agricultural sector, and Table 2 shows that 86% of the sample experienced climate variability, 8% did not, and 6% were unsure of what climate variability entails. This means that a lot of vegetable farmers are affected by climate variability, thus prompting the farmers to adopt certain strategies to mitigate the situation.

#### 3.1.2 Smart Agricultural Practices Used by the Smallholder Farmers in KwaZulu Natal

**Figure 2:**
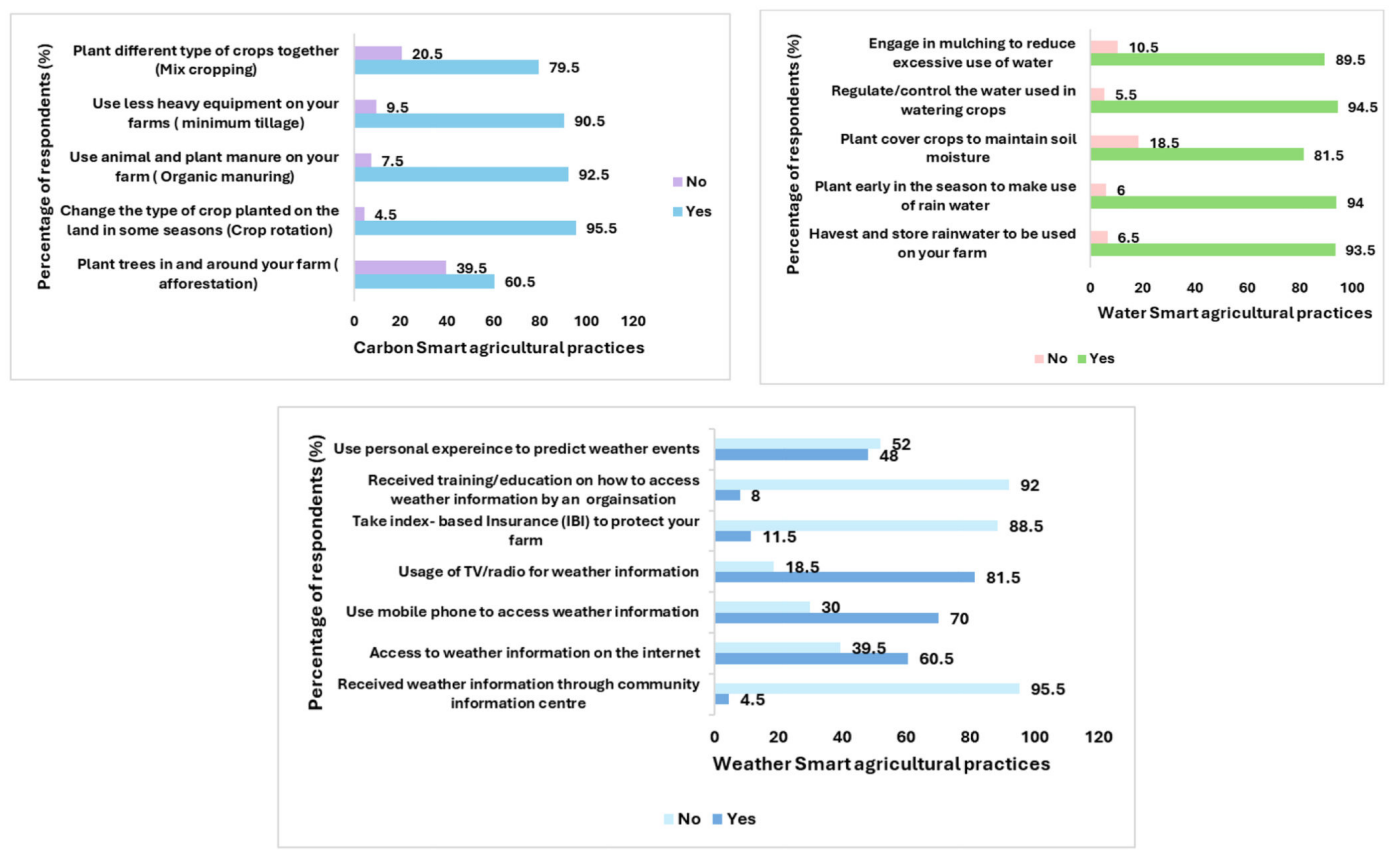
Smart agriculture practices used by smallholder farmers.

Climate-smart agricultural practices are designed to enhance the performance and resilience of smallholder farmers [41]. A significant number of farmers are engaged in seed banking (93.5%) and actively share information with colleagues (87%), reflecting their knowledge of these practices. As shown in Figure 2, many surveyed farmers utilize carbon-smart agricultural techniques such as minimum tillage, organic manure, and crop rotation. These practices aim to improve sustainability and highlight the importance of understanding climate-smart agriculture for successful adoption [42].

Figure 2 illustrates that water-smart practices are prevalent among farmers, with 94.5% controlling water use, 94% planting early to utilize rainwater, and 93.5% harvesting and storing rainwater. Employing both water- and nitrogen-smart methods is crucial for sustainable food production, mitigating the adverse effects of climate variability, and enhancing climate resilience. Integrating these practices can lead to increased crop yields, optimized resource use, and long-term agricultural sustainability [43].

Energy-smart practices are also common among smallholder farmers, with 56% composting crop residues, 32.5% using solar equipment, and 38% opting for fuel-efficient vehicles. Figure 2 shows that weather-smart practices include using TV/radio for weather updates (81.5%) and mobile phones for weather information (70%). Combining weather and energy-smart practices can enhance sustainability and productivity, helping farmers build resilience against climate variability [44].

#### 3.1.3 Reasons Why Smallholder Farmers Did Not Use Certain Adaptation Strategies in KwaZulu Natal

The findings of this study showed a widespread adoption of diverse adaptation strategies among the sampled smallholder farmers. Specifically, the data indicated high implementation rates across multiple practices: crop diversification (95%), annual crop rotation (95.5%), minimum tillage (90.5%), rainwater harvesting for irrigation (93.5%), and organic manure application (92.5%). However, the adoption of these agricultural practices was constrained by several significant barriers, including financial limitations, motivational deficits, labor constraints, methodological inefficiencies, and insufficient awareness of available strategies. Addressing these challenges necessitates a comprehensive intervention framework encompassing policy reforms, financial mechanisms, and educational initiatives [7,9]. Demographic analysis showed notable correlations between adoption rates and farmer characteristics. Age emerged as a significant determinant, with younger agricultural practitioners demonstrating a greater propensity to implement climate-smart practices compared to their older counterparts. Furthermore, educational attainment exhibited a positive association with adoption rates, as farmers with secondary and tertiary qualifications showed an increased inclination toward implementing climate-smart agricultural methodologies.

**Figure 3:**
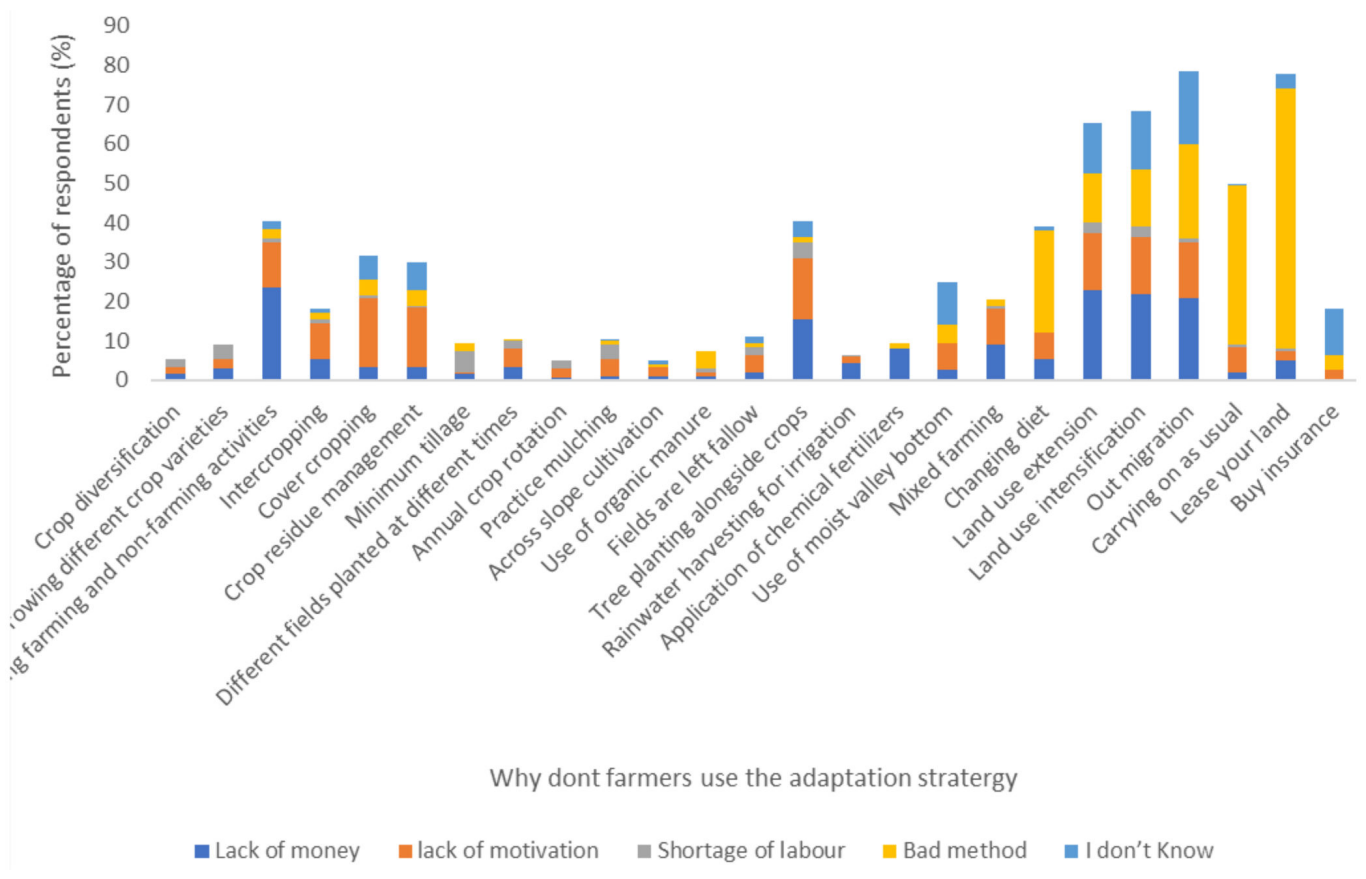
Reasons why the sampled population did not use certain adaptation strategies.

Figure 3 shows that the most common reasons for not using certain adaptation strategies were lack of motivation, ineffective methods, and financial constraints. As depicted in Figure 3 below, 15.5% of the surveyed population did not plant trees alongside their crops due to lack of money. “Lack of money” emerges as the most cited reason for not adopting strategies such as crop diversification, intercropping, cover cropping, and minimum tillage. This aligns with the established literature, suggesting that financial barriers are a critical constraint for smallholder farmers, as many strategies require upfront investment in seeds, inputs, or equipment [45]. A larger percentage (66%) of the respondents felt that leasing their land was a bad method; this is because they were scared to lose their land. Leasing property for smallholder farmers can be very detrimental because of financial stresses, power imbalances, and social ramifications [45,46]. The prevalence of financial constraints suggests that resource-limited farmers are unable to shift to more resilient practices, despite their potential to mitigate climate risks. Also, a high percentage of respondents indicated “I don’t know” as a reason for not adopting certain strategies, particularly for “Buy insurance”. This implies significant knowledge gaps regarding the existence, functioning, or benefits of agricultural insurance as a risk management tool. This finding highlights the need for targeted extension services or education campaigns to raise awareness of underutilized adaptation strategies [46,47]. It frequently results in community unrest, displacement, and decreased agricultural productivity in addition to worsening financial instability.

For strategies like “Carrying on as usual”, “Lack of motivation” is a notable barrier. This reflects a tendency among some farmers to resist change or remain reliant on traditional practices, even when they are vulnerable to climate risks. Lack of motivation often leads to reluctance to change, resulting in the use of inappropriate methods due to limited knowledge [47]. Insufficient funds restrict farmers’ ability to purchase inputs and equipment and hinder their access to credit for essential investments in adaptation measures, further exacerbating their vulnerability to climate variability [48]. These combined factors can create a cycle of challenges for smallholder farmers.

### 3.2 Empirical Results

#### 3.2.1 Determinants of Adaptation Strategies Using Extended Ordered Probit Regression

Table 3 shows the factors that influence the adaptation strategies used by the sampled smallholder farmers of KwaZulu Natal. The study focused on five adaptation strategies that were selected as they had a significant impact on the variables. These adaptation strategies were as follows: carrying on as usual, changing diet, tree planting alongside crops, diversifying farming to non-farming activities, and land use intensification. To estimate the severity of these factors on the adaptation strategies, the ordered probit regression model was used. Table 3 provides insight into the estimated results of the ordered probit regression model.

The results showed that the education level had a positive and significant influence on the “tree planting alongside crops” and “land use intensification” adaptation strategies. This implies that the higher the education level of the farmer, the more likely they were to use the adaptation strategy. The results agreed with Zamasiya et al. [49], who stated that without adequate education, smallholder farmers have lower agricultural productivity and increased susceptibility to climate hazards. This also means that if farmers have a higher education level, they are more likely to implement adaptation strategies to combat the effects of climate variability [50,51].

Education is positively associated with adopting sustainable strategies. A unit increase in education level significantly increases the likelihood of planting trees, alongside crops and moderately promotes land use intensification. Education improves farmers’ ability to understand and implement advanced techniques, including agroforestry and intensive land management practices. These findings align with Hlatshwayo et al.’s [39] existing evidence, suggesting that education enhances the adoption of climate-resilient strategies. Marital status had a negative influence on land use intensification, with significance. This means that married farmers were less likely to intensify land use due to household responsibilities and risk management priorities, making them more cautious and less inclined to adopt advanced techniques or increase inputs. This finding contrasts with the study conducted by Airflo [52], which found that land use intensity increased with marital status. However, these findings align with the study conducted by Okon et al. [53], who stated that due to household duties and risk management concerns, married farmers are less likely to intensify land usage, which makes them more conservative and less likely to embrace adaptation strategies. This relationship emphasizes the need for initiatives that address social dynamics and environmental sustainability and is influenced by household size and socioeconomic characteristics.

Household size showed a negative and significant influence on changing diet, diversifying farming to non-farming activities, and planting trees alongside crops. This implies that smallholder farmers with large household sizes limited their use of the above-mentioned adaptation strategies. This could be because when a household has more members, its resources are more depleted and thus reduce its adaptive capacity. This contradicts research by Gebre et al. [54], which states that larger household sizes increase the resilience and sustainability of the farm against climate variability. This is because larger household sizes have an increase in the labor force and, therefore, make it easier for the household to implement adaptation strategies.

The results of this study also exhibited that income had a negative and significant association with changes in diet and intensification of land use. Farming households are significantly less likely to adapt to changing diets. This finding reflects reliance on farm produce for household consumption, limiting the ability to shift dietary practices. This is a key consideration for food security policies targeting farming households. Therefore, this suggests that as smallholder farmers’ income increases, they are less likely to utilize these adaptation strategies. The findings align with Bousmaha and M’Zali [55], who observed that higher income among smallholder farmers is associated with a decreased likelihood of employing certain adaptation strategies. Generally, higher income is linked to increased resources and potentially better adoption of advanced agricultural practices. This is also contrary to Workalemahu and Dawid [9], who stated that increased income levels give smallholder farmers the means to acquire inputs, technology, and knowledge that can improve their ability to withstand the effects of climate variability.

The total size of land owned by the farmers positively and significantly affected the following adaptation strategies. Carrying on as usual: the positive and highly significant relationship suggests that farmers with larger landholdings are more likely to continue their current practices without major changes. Farmers with substantial landholdings may experience less pressure to adopt drastic adaptation strategies, as they can afford to rely on their existing practices to sustain productivity. Larger plots of land may provide a safety net by enabling diversification within the farm, thus reducing the need for visible external adaptations [56,57]. Changing diet: There is a weak significant negative relationship between landholding size and the likelihood of changing diets. Farmers with smaller landholdings are more likely to change their diets as a coping mechanism. This is likely because they rely more heavily on subsistence farming and are more exposed to food insecurity when yields drop due to climate change. Conversely, farmers with larger plots are better able to produce sufficient quantities of diverse crops for household consumption, reducing the need to adapt by changing dietary patterns. Changing diets is often a last-resort adaptation strategy, typically employed when other measures fail [56]. Smaller landholders may lack the resources to pursue other strategies, making dietary changes an unavoidable response to resource scarcity and tree planting alongside crops.

This infers that the larger the size of land owned, the farmer is more likely to employ the mentioned adaptation strategies. Studies by Kumwenda and Chirwa [56]; and Hellin et al. [57] are in line with the findings and suggest that the larger the size of land owned by farmers, the more likely and willing they are to explore different adaptation strategies to improve their crop productivity as well as overall health and increase their resistance to climate variability. The total size of land used had a negative and significant effect on the following adaptation strategies: carrying on as usual and changing diet. This proposes that the larger the land used for vegetable farming, the smaller the chance that the farmer will change their diets or carry on as usual. These findings are in line with findings from Maitra et al. [58]; and Yang et al. [59], who found a negative correlation between carrying on as usual and the size of land used for farming. These studies suggested that farmers try and reduce their risk of crop failure by using intercropping; hence, the larger the size of land that they use for crop production, the less likely the farmer is to carry on as usual.

Soil types were found to significantly and negatively influence the adaptation strategy of “carrying on as usual”. This indicates that vegetable smallholder farmers with these soil types were less likely to continue with the “carrying on as usual” approach. The findings are in agreement with Naazie et al. [11]; and Phromthep [60], who found that smallholder farmers with poor soil conditions are less inclined to adopt new adaptation strategies and tend to stick with traditional practices. This tendency arises from declining productivity, economic pressures, and limited resources, which drive farmers to rely on existing methods despite their potential unsustainability, due to the challenges related to soil fertility and the lack of viable alternatives. Farmers who had sand, silt, and loam soil had a negative and significant effect on the adaptation strategy “changing diet”. This indicates that farmers with those types of soils were reluctant to consider adopting a change in diet strategy. This means that farmers with these types of soils tend to resist changing their dietary practices, likely due to stable crop yields, economic stability, and strong cultural traditions. These findings align with studies conducted by Weil and Brady [61]; and Elias [62], who found that these factors (stable crop yields, economic stability, and strong cultural traditions) lead smallholder farmers to prefer traditional diets and familiar agricultural practices, as these generally ensure sufficient food security.

The adaptation strategy of land use intensification was negatively impacted by silt, clay, and loam soil. This suggests that farmers with silt, clay, and loam soil did not intensify their land use. These findings are in line with Xie et al. [63]; and Akanmu [64], who found that the favorable fertility and productivity of silt, clay, and loam soils, along with economic and environmental factors, lead farmers to prefer stable land use techniques over increased ones. These farmers will probably put soil health and sustainability ahead of intensifying land usage, resulting in yields that are sufficient even in the absence of extra inputs.

The results indicate that membership in farmers’ associations or cooperatives by smallholder farmers had a positive influence on adaptation strategies, such as changes in diet, diversification of farming and non-farming activities, and land use intensification. This suggests that farmers in these groups are more likely to make significant changes in these areas. According to Karlan et al. [65], which aligns with the findings, the support and resources provided by these organizations often promote stability, which may lead members to adhere to traditional practices.

Conversely, being a member of a farmers’ association or cooperative negatively and significantly impacted the use of the adaptation strategy of carrying on as usual, by the smallholder farmers. This implies that the farmers who were members of farmer’s associations or cooperatives were less likely to carry on as usual. These findings agree with Alfirdaus et al. [66]; and Magakwe and Olorunfemi [67], who stated that farmers in associations or cooperatives are more likely to adapt and innovate their practices compared to those working independently, due to the enhanced resources, information, and collective bargaining power provided by these groups. Membership in cooperatives also improves access to formal markets, better prices, and sustainable practices, fostering a collaborative culture that supports resilience and adaptability in changing agricultural conditions.

The results showed that involvement in any formal or informal food supply chain network or association by smallholder farmers had a negative and significant impact on the smallholder farmers changing their diets and diversifying their farming and non-farming activities. This indicates that farmers who engaged in such networks were less likely to adopt these adaptation strategies. This finding aligns with Garcia and Smith [68]; and Schreiber et al. [69], who noted that formal or informal food supply systems provide stability, security, and social cohesiveness, thus, making smallholder farmers within these networks less inclined to implement adaptation measures. The authors explained that this resistance to change stems from the farmers’ focus on maintaining existing connections and practices rather than exploring new approaches. Involvement in supply chains may discourage smallholder farmers in KwaZulu Natal (KZN) from adopting new adaptation strategies, as they often prioritize maintaining stable, secure networks with known partners. This conservative approach to risk management can limit their willingness to experiment with new methods or innovations that could disrupt established relationships or practices [8,68]. To address these challenges, interventions could focus on the following:

- Demonstrating how adaptation strategies can enhance rather than threaten supply chain relationships;
- Working with supply chain partners to gradually incorporate climate-smart practices.

#### 3.2.2 Factors Influencing the Level of Adaptation Strategies Among Smallholder Vegetable Farmers, KwaZulu Natal

The results of the level of implementation of the adaptation strategies and the factors that influence their usage by smallholder farmers are presented in Table 4. As shown in Table 4, the estimation of the Akaike information criterion (AIC) and Bayesian information criterion (BIC) are essential to indicate a better model in analyzing count data of the level of adoption of adaptation strategies. In this study, the Poisson regression model was used, the AIC value was 704.895, and the (BIC) value was 760.967. Household size, the total size of land owned, the total size of land used for vegetable farming, as well as sandy, silt, and loam soil were the main factors that significantly affected the level of adaptation strategy adopted by smallholder farmers in the study area. The results showed that the household size negatively and significantly influenced the adoption of the level of adaptation strategies by smallholder farmers. This indicates that smallholder farmers’ adoption of the adaptation method declines as family size grows. As was previously said, when households grow larger, people become less willing to take risks and produce primarily for their own survival rather than for the sake of selling. As a result, they are less likely to take part in interventions. The results were contrary to Adams and Mutunga [70], who stated that households with larger size of land are more likely to adapt as they can afford to make the necessary investments. According to these studies, farmers adopt more adaptation strategies to save labor and guarantee a higher crop yield as their household size increases.

The results showed that the total size of land owned by the smallholder farmers had a positive and significant influence on the level of adaptation strategy used. This indicates that the larger the size of land owned by the smallholder farmers, the higher their level of adaptation, suggesting that land size improves the household’s adaptive capacity. These findings align with studies conducted by Irawan and Syakir [13]; and Gudina and Alemu [71], who found that larger land holdings enable smallholder farmers to adopt diverse and sustainable agricultural practices, such as crop rotation and improved seed usage, which are crucial for adapting to climate variability. A study conducted by Workalemahu and Dawid [9] also aligns with the study and states that the economic stability provided by larger farms supports investment in innovative technologies and better access to markets and resources, further enhancing farmers’ ability to implement effective adaptation strategies.

The size of land used for vegetable production by smallholder farmers had a negative and significant influence on the level of adoption of adaptation strategies, which indicates that the larger the land used for vegetable production, the lower the level of adaptation strategies used by smallholder farmers. These findings contradict studies conducted by Asfaw et al. [72]; and Lengoiboni et al. [73], who found that fear of risks, financial restrictions, restricted access to resources and knowledge prevent smallholder farmers with small land sizes from using adaptation techniques. These limitations prevent the adoption of potentially helpful practices.

Lastly, sandy, silt, and loam soil showed a negative and significant influence on the level of adaptation strategy. This means that these soil types did not improve the adaptive capacity of the smallholder farmers. These results were confirmed by Verchot et al. [74]; and Keesstra et al. [75] who explained that loam, silt, and sandy soil types can have a detrimental effect on the usage of adaptation strategies by smallholder farmers. They mentioned that farmers may face unique difficulties when the proportion of these soil types rises, which makes it more difficult for them to put effective adaptation plans in place. This association emphasizes how crucial it is to take socioeconomic and soil features into account when creating policies and other measures meant to increase agricultural resilience.

## 4 Conclusions and Policy Recommendations

The implementation of adaptation techniques enhances smallholder farmers’ resilience to the effects of climate variability while also helping them to raise agricultural productivity. This study assessed the factors that influence the choice and level of adaptation strategies among smallholder vegetable farmers in KZN. The findings showed that education level and total size of land owned had a positive and significant influence on adaptation strategies used by the smallholder farmers. In contrast, marital status, household size, farming as a main source of income, type of soil, sand, silt, clay, and loam, being a member of a farmers’ association, and involvement in any formal or informal supply chain network had a negative and significant influence on the adaptation strategies. Household size, the total size of land used for vegetable production, sandy, silt, and loam soil had a significant and negative effect on the level of adaptation strategy used, whilst the total size of land owned had a positive and significant impact on the level of adaptation strategy used by the smallholder vegetable farmers.

It is concluded that socioeconomic characteristics had a considerable influence on the likelihood of employing the adaptation strategy and the level of the adaptation strategy used by the smallholder vegetable farmers in KwaZulu Natal. Education level and total land size are associated with improved farmers’ climate variability adaptative capacity. Training smallholder farmers on climate variability and adaptation methods can greatly benefit them by highlighting the importance of these strategies given current climatic conditions.

The study recommends that the government considerably supports the Climate Smart Agriculture initiatives. These may include but are not limited to alternatives like hot houses and addressing barriers through training, credit access, and sustainable practices to enhance farmers’ resilience and national food security. Additionally, overcoming barriers such as limited financial resources and motivation through targeted interventions, training, extension services, improved credit access, and sustainable practices is crucial for enhancing farmers’ resilience and ability to adapt to climate variability.

## 5 Limitations of This Study

The study acknowledges potential biases inherent in self-reported data from questionnaires and FGDs. The sample size, though sufficient for preliminary insights, may not encompass the full diversity of experiences among smallholder farmers in KwaZulu Natal. Furthermore, the four-month data collection period may not adequately capture seasonal variations and longer-term trends in climate impacts.

By employing a rigorous approach and adhering to ethical standards, this study seeks to provide a comprehensive understanding of the factors influencing the selection of adaptation strategies among smallholder vegetable farmers in rural KwaZulu Natal. The findings will offer valuable insights into the development of adaptive strategies and policies to support sustainable agricultural practices in the region. This study’s scope was limited to smallholder farmers registered with local agricultural organizations and market operators in KwaZulu Natal. While these registered farmers typically share common characteristics with the broader farming community, the findings may not fully represent unregistered smallholder farmers in the region. Future research should expand the sample to include unregistered smallholder farmers, which could provide more comprehensive insights into the entire smallholder farming sector in KwaZulu Natal. This broader inclusion would help validate the current findings and potentially identify unique challenges or needs specific to unregistered farmers.

## Figures and Tables

**Table 1:** Description of variables (own source).

Variable	Description of Variable	Expected Outcome
Gender	Dummy (1 = Male, 2 = Female)	−
Age	Measured in numbers	−
Education level	Continuous (1 = No formal schooling, 2 = Primary, 3 = Secondary, 4 = Tertiary and above)	+
Marital status	Continuous (1 = Single, 2 = Married, 3 = Divorced, 4 = Widowed)	+
Household size	Measured in numbers	+
Number of years farming—vegetables	Measured in numbers	+
Is farming your main source of income?	Dummy (1 = Yes, 2 = No)	+
Total size of land owned (hectare)	Measured in numbers	+
Total size of land used for Farming.	Measured in numbers	+
Type of soil—Sand	Dummy (1 = Yes, 2 = No)	−
Type of soil—Silt	Dummy (1 = Yes, 2 = No)	+
Type of soil—Clay	Dummy (1 = Yes, 2 = No)	−
Type of soil—Loam	Dummy (1 = Yes, 2 = No)	+
Are you a member of a farmers’ association/co-operation?	Dummy (1 = Yes, 2 = No)	+
Involved in any formal or informal food supply chain networks or associations	Dummy (1 = Yes, 2 = No)	+

Source: Survey data.

**Table 2:** Demographics of the surveyed population.

Variable		Frequency (n = 200)	Percentage (%)
Gender	Male	130	65
	Female	70	35
Education level of respondents	No Formal Education	12	6
	Primary	50	25
	Secondary	101	50.5
	Tertiary and above	37	18.5
Household Size	1–2	53	26.5
	3–4	75	37.5
	5–6	37	18.5
	7–8	14	7
	9–10	11	5.5
	11–12	8	4
	13–14	2	1
Age	26–39	45	22.5
	40–53	60	30
	54–67	56	28
	68–81	34	17
	82–88	5	2.5
Is farming the main source of income	Yes	170	85
	No	30	15
Part of a:	Farmers association	66	33
	Cooperation	53	26.5
	Not applicable	81	40.5
Did the respondent experience climate variability	Yes	172	86
	No	16	8
	I do not Know	12	6

**Table 3:** Determinants of adaptation strategies using extended ordered probit regression.

Variable	Carrying on as Usual			Changing Diet			Tree Planting Alongside Crops			Diversifying Farming to Non-Farming Activities			Land Use Intensification		
	Coefficient	St. Errs.	*p*-Value	Coefficient	St. Errs.	*p*-Value	Coefficient	St. Errs.	*p*-Value	Coefficient	St. Errs.	*p*-Value	Coefficient	St. Errs.	*p*-Value
Gender	−0.208	0.239	0.385	0.120	0.228	0.600	0.146	0.217	0.502	−0.081	0.209	0.698	0.137	0.228	0.549
Age	−0.010	0.012	0.405	−0.003	0.012	0.813	0.011	0.011	0.350	−0.010	0.011	0.377	0.019	0.012	0.108
Education Level	0.191	0.165	0.246	−0.263	0.160	0.100	0.302	0.149	0.042 **	0.168	0.150	0.261	0.286	0.162	0.077 *
Marital status	−0.103	0.148	0.485	−0.026	0.145	0.860	0.059	0.135	0.665	0.015	0.130	0.910	−0.333	0.154	0.031 **
Household size	0.034	0.043	0.426	−0.083	0.042	0.050 **	−0.070	0.040	0.081 *	−0.091	0.040	0.025 **	−0.040	0.045	0.383
Number of years farming—vegetables	0.019	0.013	0.127	−0.012	0.012	0.345	−0.008	0.012	0.514	0.007	0.011	0.530	−0.009	0.012	0.450
Is farming your main source of income?	0.368	0.330	0.264	−0.909	0.313	0.004 ***	−0.317	0.280	0.258	−0.091	0.285	0.748	−0.599	0.321	0.062 *
Total size of land owned (hectare)	0.038	0.012	0.001 ***	0.018	0.011	0.098 *	0.019	0.010	0.064 *	0.007	0.010	0.471	0.015	0.010	0.126
Total size of land used for Farming.	−0.173	0.041	0.000 ***	−0.183	0.035	0.000 ***	−0.031	0.023	0.165	0.003	0.023	0.901	0.025	0.023	0.276
Type of soil—Sand	−1.131	0.272	0.000 ***	−0.848	0.282	0.003 ***	0.147	0.242	0.544	−0.279	0.238	0.241	−0.074	0.246	0.765
Type of soil—Silt	−0.841	0.286	0.003 ***	−0.992	0.292	0.001 ***	0.173	0.261	0.507	−0.333	0.260	0.200	−0.632	0.279	0.023 **
Type of soil—Clay	−0.576	0.293	0.049 **	−0.384	0.292	0.189	−0.217	0.263	0.410	0.269	0.254	0.289	−0.456	0.259	0.078 *
Type of soil—Loam	−0.576	0.267	0.031 **	−0.883	0.283	0.002 ***	−0.050	0.255	0.845	0.078	0.251	0.755	−0.487	0.265	0.066 *
Are you a member of a farmers’ association/co- operation?	−0.151	0.087	0.082 *	0.271	0.086	0.002 ***	0.112	0.075	0.138	0.190	0.076	0.012 **	0.148	0.079	0.060 *
Involved in any formal or informal food supply chain networks or associations	0.200	0.251	0.426	−0.586	0.252	0.020 ***	0.158	0.216	0.466	−0.555	0.229	0.015 **	−0.426	0.234	0.069 *
Cons	4.883	1.795	0.007 ***	9.257	2.030	0.000 ***	−1.548	1.617	0.338	1.386	1.629	0.395	2.033	1.704	0.233

*** *p* < 0.01,

** *p* < 0.05,

* *p* < 0.1.

**Table 4:** Factors influencing the level of adaptation strategies among smallholder vegetable farmers, KZN.

Variable	Poisson			Marginal Effect		
	Coef.	St. Err.	*p*-Value	dy/dx	Std.	*p*-Value
Gender of respondent	0.008	0.099	0.939	0.019	0.249	0.939
Age of respondent	−0.001	0.005	0.906	−0.002	0.013	0.906
Education level	0.083	0.071	0.247	0.208	0.179	0.246
Marital status	−0.014	0.061	0.822	−0.034	0.152	0.822
Household size	−0.038	0.020	0.056 *	−0.096	0.050	0.055 *
Number of years farming (vegetable)	0.000	0.005	0.978	−0.000	0.013	0.978
Is farming your main source of income	−0.125	0.130	0.336	−0.314	0.326	0.336
Total size of land owned (hectare)	0.009	0.004	0.030 **	0.022	0.010	0.029 **
Total size of land used for vegetable production	−0.029	0.012	0.015 **	−0.073	0.030	0.014**
Type of soil—Sand	−0.260	0.113	0.021 **	−0.653	0.282	0.021 **
Type of soil—Silt	−0.251	0.117	0.032 **	−0.631	0.293	0.031 **
Type of soil—Clay	−0.132	0.116	0.256	−0.332	0.291	0.255
Type of soil—Loam	−0.227	0.112	0.042 **	−0.571	0.281	0.042 **
Are you a member of a farmers’ association/co-operation	0.051	0.034	0.138	0.127	0.086	0.137
Involved in any formal or informal food supply chain networks or associations	−0.096	0.105	0.362	−0.241	0.265	0.362
Constant	2.540	0.739	0.001 ***			***
Mean dependent var	2.625					
Pseudo r-squared	0.064					
Chi-square	46.117					
Akaike crit. (AIC)	704.895					
Bayesian crit. (BIC)	760.967					
SD dependent var	1.433					
Number of obs	200.000					
Prob > chi2	0.000					

*** *p* < 0.01,

** *p* < 0.05,

* *p* < 0.1.

## Data Availability

Data are available on request from the authors.

## References

[1] Zheng X, Liu M (2019). Trade-offs and synergies in ecosystem services for sustainability. Front Environ Sci.

[2] Baselice A, Prosperi M, Lopolito A (2020). A conceptual framework for the evaluation of social agriculture: An application to a project aimed at the employability of young people need. Sustainability.

[3] Tian T, Li L, Wang J (2022). The effect and mechanism of agricultural informatization on economic development: Based on a spatial heterogeneity perspective. Sustainability.

[4] Nodayizana A, Ritter K (2022). Assessing the effectiveness of government-funded smallholder development projects in the eastern cape, south africa: The case of the raymond mhlaba municipality. Stud Mundi—Econ.

[5] STATSA (2022). https://www.statssa.gov.za/publications/P1101/P11012022.pdf.

[6] Chisasa J (2020). Determinants of access to bank credit by smallholder farmers: Evidence from South Africa. Int J Econ Bus Financ.

[7] Mufudza M, Gukurume S (2021). Perceptions of smallholder farmers on climate change impacts and adaptation strategies in rural Limpopo Province, South Africa. Clim Dev.

[8] Naranjo L, Torres J, Pérez H, Mendoza G (2020). The role of policy frameworks in climate change adaptation among smallholder farmers in sub-Saharan Africa. J Environ Econ Manag.

[9] Workalemahu S, Dawid I (2021). Smallholder farmers’ adaptation strategies, opportunities and challenges to climate change: A review. Int J Food Sci Agric.

[10] Zeleke T, Beyene F, Deressa T, Yousuf J, Kebede T (2022). Smallholder farmers’ perception of climate change and choice of adaptation strategies in east hararghe zone, eastern ethiopia. Int J Clim Chang Strateg Manag.

[11] Naazie GK, Dakyaga F, Derbile EK (2023). Agro-ecological intensification for climate change adaptation: Tales on soil and water management practices of smallholder farmers in rural Ghana. Discov Sustain.

[12] Sitaula BK, Muringai V, Mulugetta Y (2021). Determinants of smallholder farmers’ adaptation strategies to the effects of climate change: Evidence from northern Uganda. Agric Food Secur.

[13] Irawan A, Syakir M (2019). Determinants of oil palm smallholder farmers’ adaptation strategy to climate change in bengkulu, indonesia. Rev Econ Sociol Rural.

[14] Myeni L, Moeletsi M (2020). Factors determining the adoption of strategies used by smallholder farmers to cope with climate variability in the eastern free state, south Africa. Agriculture.

[15] Olabanji M, Davis N, Ndarana T, Kuhudzai A, Mahlobo D (2021). Assessment of smallholder farmers’ perception and adaptation response to climate change in the olifants catchment, South Africa. J Water Clim Chang.

[16] Donatti CI, Harvey CA, Martínez-Rodríguez MR, Vignola R, Rodríguez CM (2019). Vulnerability of smallholder farmers to climate change in Central America and Mexico: Current knowledge and research gaps. Clim Dev.

[17] Vignola R, Martínez-Rodríguez M, Saborío-Rodríguez M, Harvey C, Donatti C (2021). Climate change and adaptation strategies for smallholder farmers in Central America and Mexico: Knowledge gaps and needs for policy development. Agric Syst.

[18] Adeagbo O, Ojo T, Adetoro A (2021). Understanding the determinants of climate change adaptation strategies among smallholder maize farmers in south-west, nigeria. Heliyon.

[19] Hirpha H, Mpandeli S, Bantider A (2020). Determinants of adaptation strategies to climate change among the smallholder farmers in adama district, ethiopia. Int J Clim Chang Strateg Manag.

[20] Addis Y, Abirdew S (2021). Smallholder farmers’ perception of climate change and adaptation strategy choices in central ethiopia. Int J Clim Chang Strateg Manag.

[21] Ikua M (2021). Constraints and opportunities for greenhouse farming technology as an adaptation strategy to climate variability by smallholder farmers of nyandarua county of kenya. East Afr J Sci Technol Innov.

[22] Mutekwa V, Moyo C, Mashingaidze K (2020). Assessing climate change impacts and adaptation strategies of smallholder farmers in Zimbabwe: A case study in the Zambezi Valley. J Agric Rural Dev.

[23] Mwangi H, Kamau J, Gikonyo M (2019). Assessing adaptation strategies of smallholder vegetable farmers to climate change in Kenya. J Clim Chang Agric.

[24] Municipalities, K.N (2023). Municipal Directories and Reports.

[25] South African Department of Agriculture, Forestry and Fisheries (2021). Impact of Climate Change on Agricultural Production in KwaZuluNatal.

[26] Smith J, Brown L (2020). The impact of flash floods and extreme heat on smallholder farmers in KwaZulu-Natal. Agric Syst.

[27] Arif Ahmad D, Mahrinasari M, Dwi ASA (2023). The influence of product packaging design and social media advertising on purchase intention. Brill Int J Manag Tour.

[28] Morgan G, Parker S (2024). How Multisensory Environments Help Reduce Anxiety for Students.

[29] Asrial A, Syahrial S, Kurniawan DA, Chen D, Wulandari M (2022). E-module mangrove ecotourism: Difference and relationship perception, interest, and environment character care elementary students. J Ilm Peuradeun.

[30] Tanaka T, Miki K (2022). Random sampling methodologies for representative population surveys without complete sampling lists: A comparative analysis. J Soc Res Methods.

[31] Beharielal T, Thamaga-Chitja J, Schmidt S (2022). Socioeconomic Characteristics Associated with Farming Practices, Food Safety and Security in the Production of Fresh Produce—A Case Study including Small-Scale Farmers in KwaZulu-Natal (South Africa). Sustainability.

[32] Tzortzios S, Gitsakis N, Adam GK (2019). Management of huge amounts of data using qualitative and statistical modeling: An agricultural case study. Biom Biostat Int J.

[33] Abadi T, Kidane H, Melaku T, Gebretsadik D, Hagos H, Teklay Z, Kelelew H (2020). Determinants in utilizing improved agricultural technologies for enhancing sorghum production in tigray region, northern ethiopia. Int J Agric Ext Soc Dev.

[34] Hiko M, Mosisa W, Dinku A (2020). Determinants of adoption of agricultural extension package technologies by smallholder households on sorghum production: Case of gemechis and mieso districts of west hararghe zone, oromia regional state, ethiopia. J Agric Ext Rural Dev.

[35] Zhang Y, Zhang Y (2021). Marginal effects in multivariate probit and multinomial models: An application in healthcare economics. J Health Econ.

[36] Giroh YD, Nachandiya N (2020). A poisson regression analysis of COVID-19 pandemic: Implication on food security in Northeastern Nigeria. SSRN.

[37] Abate D, Addis Y (2021). Factors affecting the intensity of market participation of smallholder sheep producers in northern Ethiopia: Poisson regression approach. Cogent Food Agric.

[38] Chekol F, Hiruy M, Tsegaye A, Mazengia T, Alimaw Y (2022). Consumers’ frequency of purchasing behavior of organic honey and butter foods from the farmers’ food product market in Northwest, Ethiopia: A poisson regression approach. Cogent Soc Sci.

[39] Hlatshwayo S, Ngidi M, Ojo T, Modi A, Mabhaudhi T, Slotow R (2021). A typology of the level of market participation among smallholder farmers in South Africa: Limpopo and Mpumalanga provinces. Sustainability.

[40] Phakathi M, Moyo P, Dube S (2021). The role of farmer groups in enhancing fertiliser use among smallholder farmers in rural South Africa: Evidence from KwaZulu-Natal. Afr J Agric Res.

[41] Chavula P (2024). Factors influencing climate-smart agriculture practices adoption and crop productivity among smallholder farmers in Nyima district, Zambia. F1000Research.

[42] Shani F (2024). Determinants of smallholder farmers’ adoption of climate-smart agricultural practices in zomba, eastern malawi. Sustainability.

[43] Gichuki C, Osewe M, Ndiritu S (2023). Dissemination of climate smart agricultural knowledge through farmer field schools (ffs): Analyzing the application cas knowledge by smallholder farmers. Int J Dev Issues.

[44] Awazi A (2022). Agroforestry for climate change adaptation, resilience enhancement and vulnerability attenuation in smallholder farming systems in cameroon. J Atmos Sci Res.

[45] Timmermann C, Félix G (2019). Sustainable Governance and Management of Food Systems.

[46] Vu HT, Goto D (2020). Does awareness about land tenure security (LTS) increase investments in agriculture? Evidence from rural households in Vietnam. Land Use Policy.

[47] Hoffmann V, Mude A, Villano R (2019). Barriers and enablers to climate adaptation in sub-Saharan Africa: A systematic review. Environ Sci Policy.

[48] Oduniyi O, Sylvia T (2019). Establishing the nexus between climate change adaptation strategy and smallholder farmers’ food security status in south africa: A bi-casual effect using instrumental variable approach. Cogent Soc Sci.

[49] Zamasiya B, Nyikahadzoi K, Mukamuri B (2020). African Handbook of Climate Change Adaptation.

[50] Kom Z, Nethengwe N, Mpandeli N, Chikoore H (2020). Determinants of small-scale farmers’ choice and adaptive strategies in response to climatic shocks in vhembe district, south africa. GeoJournal.

[51] Kurniawati N, Luvhengo U (2021). Defining Indonesian and African Small-Holder Farmers’ Climate Change Adaptive Capacity and Practices: A Brief Argument.

[52] Arifalo SF (2024). Determinants of land-use intensity among cassava farmers in Ondo state, Nigeria. Discov Agric.

[53] Okon U, Essien U, Udousoro I (2019). Drivers of households’ decision making in agroforestry practices in Akwa Ibom state, Nigeria. Adv Soc Sci Cult.

[54] Gebre G, Amekawa Y, Ashebir A (2023). Can farmers’ climate change adaptation strategies ensure their food security? Evidence from Ethiopia. Agrekon.

[55] Bousmaha F, M’Zali S (2023). Microfinance, financial self-sufficiency, and agricultural technology adoption in rural economies. Int J Dev Sustain.

[56] Kumwenda PA, Chirwa RW (2023). Gender roles in climate change adaptation strategies: A case study of rural Malawi. J Rural Stud.

[57] Hellin J, Fisher M, Tambo JA (2023). Climate-Smart Agriculture and the Resilience of Smallholder Farming Systems in Sub-Saharan Africa: A Review. Agric Food Secur.

[58] Maitra S, Hossain A, Brestič M, Skalický M, Ondrišík P, Gitari HI, Sairam M (2021). Intercropping—A low input agricultural strategy for food and environmental security. Agronomy.

[59] Yang P, Cai X, Khanna M (2021). Farmers’ heterogeneous perceptions of marginal land for biofuel crops in us midwestern states considering biophysical and socioeconomic factors. GCB Bioenergy.

[60] Phromthep P, Torut B (2024). Comparing collaboration of smallholder farmers through participatory guarantee system practices in northeastern Thailand. Sustainability.

[61] Weil RR, Brady NC (2021). This updated edition reflects the most recent advancements in soil science, including new discussions on soil health, sustainable practices, and the role of soils in mitigating climate change. Nat Prop Soils.

[62] Elias M (2023). The diasporic meatscapes of the Tamil community in Toronto: How immigrants reconfigure food environments and infrastructures to secure a taste of home. Food Cult Soc.

[63] Xie H, Huang Y, Chen Q, Zhang Y, Wu Q (2019). Prospects for agricultural sustainable intensification: A review of research. Land.

[64] Akanmu O (2023). Agroecology and sustainable intensification of smallholder farming systems in Sub-Saharan Africa. Front Sustain Food Syst.

[65] Karlan D, Thuysbaert B, Parienté W, Osei R, Shapiro J, Duflo E, Banerjee A, Goldberg N, Udry C (2020). Long-term effects of the Graduation program on extreme poverty: Evidence from six countries. Am Econ J Appl Econ.

[66] Alfirdaus LK, Manalu SPB, Ardianto HT, Kushandajani K (2020). Proceedings of the 5th International Conference on Indonesian Social and Political Enquiries, ICISPE 2020, Semarang, Indonesia, 9–10 October 2020.

[67] Magakwe T, Olorunfemi F (2024). The role of collective marketing in smallholder agricultural entrepreneurship: A systematic review across Sub-Saharan Africa. J Agric Econ Dev.

[68] Garcia R, Smith R (2021). Climate-smart agriculture and food systems in Africa: A review of the state of knowledge and future research directions. J Agric Syst.

[69] Schreiber K, Soubry B, Dove-McFalls C, MacDonald G (2022). Diverse adaptation strategies helped local food producers cope with initial challenges of the covid-19 pandemic: Lessons from québec, canada. J Rural Stud.

[70] Adams R, Mutunga C (2019). Assessing Climate Change Impacts on Agriculture and Food Security in Sub-Saharan Africa: Adaptation Strategies for Maize Farmers. Agric Syst.

[71] Gudina MH, Alemu EA (2024). Factors influencing smallholder farmers’ adoption of climate-smart agriculture practices in Welmera Woreda, Central Ethiopia. Front Clim.

[72] Asfaw S, Shiferaw B, Simtowe (2020). Agricultural technology adoption, seed access constraints, and commercialization in Ethiopia. Agric Econ.

[73] Lengoiboni M, Zevenbergen J, Simane B (2023). Rethinking the impact of land certification on tenure security, land disputes, land management, and agricultural production: Insights South Wello Ethiop. Land.

[74] Verchot LV, Dannenmann M, Kengdo SK, Njine-Bememba CB, Rufino MC, Sonwa DJ, Tejedor J (2020). Land-use change and Biogeochemical controls of soil CO2, N2O and CH4 fluxes in Cameroonian forest landscapes. J Integr Environ Sci.

[75] Keesstra SD, Bouma J, Goode JR (2021). The role of soils in achieving the sustainable development goals: A review. Soil.

